# Severe asthma in childhood

**DOI:** 10.1186/1824-7288-41-S2-A23

**Published:** 2015-09-30

**Authors:** Fernando Maria de Benedictis

**Affiliations:** 1 Dipartimento Materno-Infantile, Azienda Ospedaliero-Universitaria, Ospedali Riuniti, Ancona, Italy

## 

In the majority of cases, asthma in children may be easily controlled by available drugs. However, a substantial subset exists who remain “difficult to treat”. The burden of the disease for these children is enormous in terms of quality of life, social problems, resources expenditure and mortality.

Over the last 15 years, several international committees have proposed different definitions for severe asthma. In order to revise such definition and provide guidance about the management of patients with severe asthma, a jointed ERS/ATS Task Force has recently published a document addressed to the specialists in respiratory medicine and allergy [1].

It is defined “severe”, asthma which requires treatment with guidelines suggested medications for GINA steps 4–5 asthma (high dose ICS and LABA or leukotriene modifier/theophylline) for the previous year or systemic CS foro 50% of the previous year to prevent it from becoming “uncontrolled” or which remains “uncontrolled” despite this therapy.

Uncontrolled asthma defined as at least one of the following: 1) Poor symptom control: ACQ consistently >1.5, ACT <20 (or “not well controlled” by NAEPP/GINA guidelines); 2) Frequent severe exacerbations: two or more bursts of systemic CS (>3 days each) in the previous year; 3) Serious exacerbations: at least one hospitalisation, ICU stay or mechanical ventilation in the previous year; 4) Airflow limitation: after appropriate bronchodilator withhold FEV1 <80% predicted (in the face of reduced FEV1/FVC defined as less than the lower limit of normal); 5) Controlled asthma that worsens on tapering of these high doses of ICS or systemic CS (or additional biologics).

Inherent in the definition of severe asthma is the exclusion of individuals who present with “difficult asthma” in whom appropriate diagnosis and/or treatment of confounders (comorbidity, adherence, psychosocial problems, etc.) improves their current condition. Therefore, it is recommended that patients presenting with “difficult asthma” have their asthma diagnosis confirmed and be evaluated by an asthma specialist for more than 3 months.

Patients with confirmed severe asthma should receive an individualised treatment plan after a detailed and invasive protocol of investigations. Therapeutic options can be divided into medications used in lower doses for children with less severe asthma, and those used in other paediatric diseases but not for asthma. Most treatments are unlicensed and the evidence base is poor. International collaborations, using standard protocols of investigation, will be essential if the mechanisms of severe therapy resistant asthma are to be understood, and evidence-based treatment delivered.
